# Shrimp Lipids: A Source of Cancer Chemopreventive Compounds

**DOI:** 10.3390/md11103926

**Published:** 2013-10-16

**Authors:** Carmen-María López-Saiz, Guadalupe-Miroslava Suárez-Jiménez, Maribel Plascencia-Jatomea, Armando Burgos-Hernández

**Affiliations:** Department of Research and Food Science Graduate Program, University of Sonora, Apartado Postal 1658, Hermosillo, Sonora 83000, México; E-Mails: k_rmelita@hotmail.com (C.-M.L.-S.); msuarez@guayacan.uson.mx (G.-M.S.-J.); mplascencia@guayacan.uson.mx (M.P.-J.)

**Keywords:** shrimp, chemoprevention, fatty acids, carotenoids, cancer

## Abstract

Shrimp is one of the most popular seafoods worldwide, and its lipids have been studied for biological activity in both, muscle and exoskeleton. Free fatty acids, triglycerides, carotenoids, and other lipids integrate this fraction, and some of these compounds have been reported with cancer chemopreventive activities. Carotenoids and polyunsaturated fatty acids have been extensively studied for chemopreventive properties, in both *in vivo* and *in vitro* studies. Their mechanisms of action depend on the lipid chemical structure and include antioxidant, anti-proliferative, anti-mutagenic, and anti-inflammatory activities, among others. The purpose of this review is to lay groundwork for future research about the properties of the lipid fraction of shrimp.

## 1. Introduction

Shrimp is one of the most popular seafoods of traditional diets [1] worldwide, and the top ten shrimp-producing nations include some of the richest and poorest nations in the world [2]. World shrimp production has increased in the last few years, from 2.85 up to 3.12 million tons (2002 and 2008, respectively) [3]. Shrimp muscle is rich in high quality proteins and minerals, and is low in fat content [1,4]; in addition, its lipids exhibit chemopreventive and chemoprotective activities, which are important biological properties in thin product.

Several biological activities have been reported for methanolic and lipidic extracts from shrimp muscle [5,6] and waste [7]. These activities, which are capable of modifying biological processes [8], have been related to cancer prevention throughout mechanisms grouped in a term called chemoprevention, a term that is used to describe the use of natural or synthetic substances to prevent cancer development [9]. Cancer, the leading cause of death in economically developed countries and second in developing countries [10], affects approximately one of three individuals in Europe and in the United States of America, appearing as one of one hundred different kinds of this disease, with a mortality rate of approximately 20% [11]. By the year 2020, world population is expected to increase up to 7.5 billion, and approximately 17 million new cancer cases will be diagnosed [12].

In addition to socioeconomic status [13,14], geographic variability [13,15], age [16], and physical activity [13,17], nutrition is one of the factors that may influence the development of cancer and other human diseases. Nowadays, changes in the life style that include the consumption of chemopreventive compounds, such as those found in a great variety of foods, are highly recommended. In this review, the mechanisms of action of compounds that are found in a very popular seafood such as shrimp, especially in its lipidic fraction, will be discussed.

## 2. Chemoprevention

### 2.1. Definition of Chemoprevention

Epidemiological studies have provided convincing evidence that naturally occurring bioactive extracts or isolated compounds may benefit human health through the inhibition of carcinogenic processes and cell death mechanisms [18,19]. New technologies and genetic engineering have accounted for unlimited possibilities in scientific discoveries, which have raised a potential for a number of health beneficial products such as chemopreventive compounds [20]; this constitutes an area of research in disease prevention [21]. Chemoprevention was originally defined by Sporn (1976) as the use of natural, synthetic, or biologic chemical agents, in order to reverse, suppress, or prevent cancer progression [9]. Chemoprevention strategies address four goals: inhibition of carcinogens, logical intervention in persons at genetic risk, treatment of pre-malignant lesions, and translation of leads from dietary epidemiology to intervention strategies. Agents that may be useful to achieve at least one of these goals are broadly classified into three categories: blocking agents, suppressing agents, and those that reduce tissue vulnerability to carcinogenesis [22,23].

### 2.2. Types of Chemopreventive Activities

Chemopreventive compounds can be subdivided according to the benefit they offer to human health; among those are antioxidant, antimutagenic, antiproliferative, antiinflamatory, and antiangiogenic.

Antioxidant chemopreventive compounds prevent or delay oxidation at low concentrations, offering protection against oxidation mainly due to free radicals [24], molecules that are characterized by high reactivity due to non-paired electrons of external orbitals in some of their atoms. Free radicals have mechanisms of action that harm cells and body tissues, damage proteins, DNA, and lipids [25]. Antioxidants prevent cellular damage by reacting with oxidizing free radicals and promoting their elimination from the organism; these free radicals may be present in the cell at an oxidative stress event or during an event induced by an external source such as chemical compounds or ionizing radiation (including singlet oxygen, hydroxyl radical, peroxyl radicals, superoxide anion, hydrogen peroxide, nitric oxide, among others). Antioxidants can be found in foods as micro and macronutrients and may be able to promote the regression of premalignant lesions and inhibit their development into a cancer [26].

Antimutagenic chemopreventive compounds offer protection against cell DNA mutation caused by mutagenic agents (that alters the DNA) and slow cancer initiation [27], while antiproliferative compounds interfere in the cell cycle preventing and/or slowing down uncontrolled cancer cell division.

Inflammation promotes cell proliferation and is linked to carcinogenesis. Although proliferation alone does not cause cancer, a sustained proliferation in an environment rich in inflammatory cells, growth factor, activated stroma, and DNA-damage-promoting agents, potentiates and/or increases neoplastic risk [28]. Anti-inflammatory compounds might help to prevent the development of suitable environments for tumors [21]. Finally, antiangiogenic compounds prevent proliferation of cancerous cells by reducing the amount of blood nutrients in the tumor environment. Angiogenesis, described as the formation of new blood vessels that allow sustained tumor growth [29], is the result of loss balance between pro- and anti-angiogenic factors.

**Figure 1:**
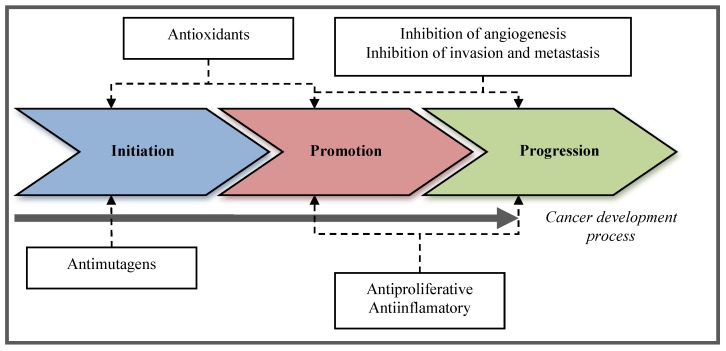
Stages during cancer development where chemopreventive compounds exert their activity.

Molecules with these activities are directed to one or more cancer stages, including anti-initiation, anti-promotional, and anti-progression strategies (Figure 1). Nature is a vast source of novel therapeutic compounds with diverse origins in plants, animals, and marine species, as well as from microorganisms that have been also reported as chemopreventive. Most chemopreventive compounds have been found in fruits and vegetables [30,31]; however, the marine environment, due to its extensive biodiversity, is a rich source of biological active compounds, such as lipids, sterols, proteins, polysaccharides, among others [32,33,34] yet to be discovered and studied.

## 3. Chemopreventive Compounds in the Lipidic Fraction of Shrimp

More than 15,000 natural compounds and extracts have been obtained from marine organisms [35] and have been tested for biological activities. These include compounds such as peptides and depsipeptides, extracted from tunicates, sponges, and mollusks [36]; shark’s cartilage [37], and squalamine [38], obtained from the squalidae family; lipidic extracts, isolated from yellowtail fish [39], shrimp [5], and octopus [40]; marine pigments, among others. Some of these, such as carotenoids, appear to fit the criteria for the development of functional food ingredients [34]. Contribution of the marine environment to therapeutic approaches for cancer and other chronic-degenerative diseases are expected to increase in the future [41]. Shrimp is a marine organism in which chemopreventive molecules have been detected, such as lipids and lipophylic compounds. Wilson-Sanchez *et al*. (2010) [5] demonstrated that several compounds in the lipidic fraction of shrimp muscle have antimutagenic and antiproliferative activities. Also, Sindhu and Sherief (2011) [42] proved antioxidant and antiinflamatory activities in carotenoids and lipids obtained from shrimp shell. Different compounds integrate the lipidic fraction of shrimp and their mechanisms of action are diverse, which mainly depend on their chemical structures; these issues will be discussed in the subsequent sections.

### 3.1. Lipidic Content of Shrimp Muscle

The lipid fraction represents the 1%–2% [43] of shrimp muscle weight (dry basis) and it is integrated by carotenoids, phospholipids, neutral lipids (including cholesterol, triglycerides, free fatty acids, diglycerides, and monoglycerides) and glycolipids. Carotenoids have been studied for chemopreventive properties and they constitute the main group of pigments found in aquatic animals [44] producing colors from yellow to dark red [45]. The main chain of their chemical structure is 40 carbon long, highly unsaturated, inflexible, and easily oxidizable [46], most of them symmetrical around the central carbon atom. These pigments are lipophylic and those with polar hydroxyl and keto functionalities have increased affinities for lipid/water interfaces [47,48]. To date, more than 750 carotenoids have been identified in nature and over 250 of those are from marine origin [49]; nevertheless only 24 have been identified in human tissues.

Fatty acids, known as any aliphatic monocarboxylic acid that may be released by hydrolysis of natural fat [50], have a carboxyl group at the head end and a methyl group at the tail end [51], and constitute the starting point in many lipid structures [52]. Fatty acids can be classified as saturated, monounsaturated, and polyunsaturated, according to the number of double bounds in their structure. The polyunsaturated compounds are characteristic in marine animals. Their mechanisms of action involved in cancer chemoprevention are discussed in the next section.

### 3.2. Carotenoids

Carotenoids are synthesized by members of the plant kingdom and they are transferred to animals through the food chain [48]. The industrial use of these compounds for animal feed supplementation has been suggested in order to enhance the pigmentation of fish skin and flesh, and also as a human nutraceutical [53]. Since various natural carotenoids (such as zeaxanthin, lycopene, β-cryptoxanthin, fucoxanthin, astaxanthin, capsanthin, crocetin, and phytoene), have proven to have anticarcinogenic activity, they have been proposed for cancer treatment and bio-chemoprevention [54] at concentrations obtained from food supply. Higher concentrations have been related to adverse effects on cellular function, and even augment cancer risk [55].

β-Carotene and cantaxanthin have proved chemopreventive activity in induced skin cancer in female Swiss albino mice [56]; these carotenoids have a protective effect against indirectly-induced skin and breast cancer, and also against directly-induced gastric carcinogenesis [57]. Thus, many studies have been focused on proving that dietary carotenoids are in fact chemopreventive agents, highly valued information that could be used for the benefit of general population.

**Figure 2:**
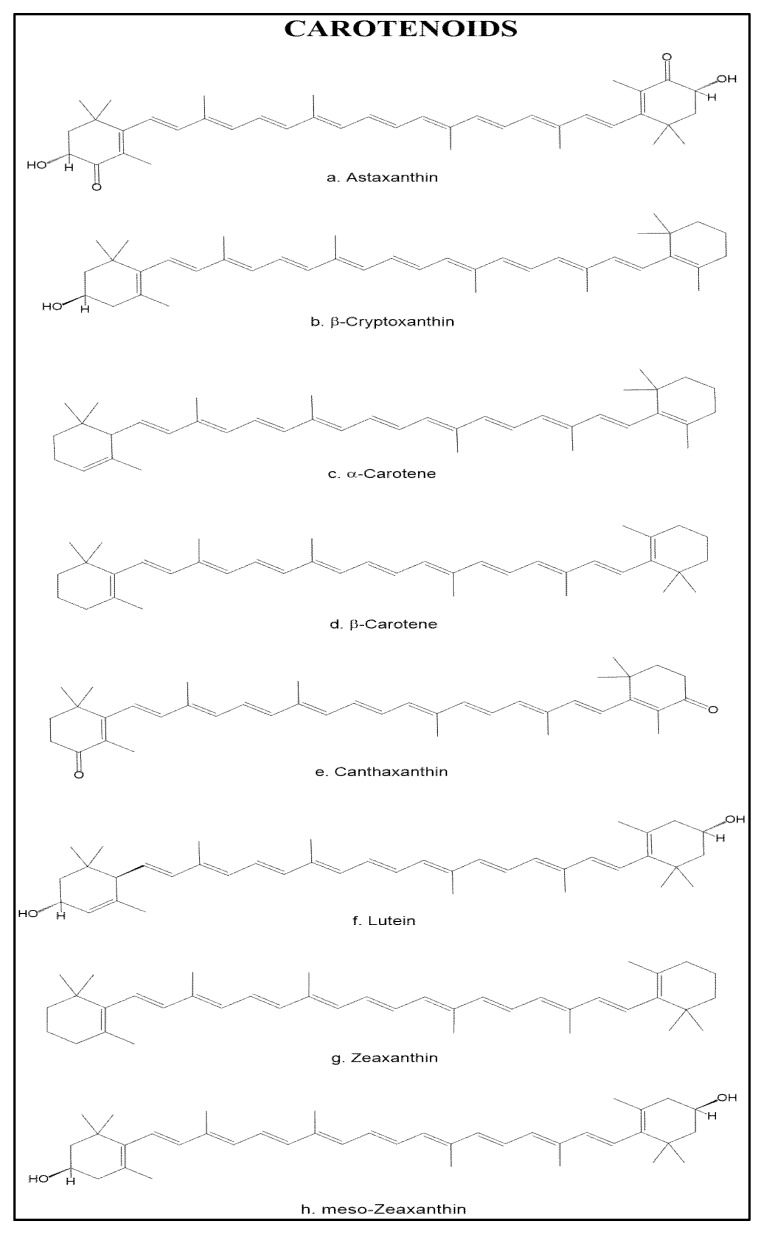
Chemical structure of the main carotenoids found in shrimp.

Shrimp is a source of different carotenoids; the main one found in the Penaeidae family is astaxanthin, a keto-oxycarotenoid that contains oxygen functional groups on the lateral rings, which classifies it among the xanthophylls. This carotenoid is found in high amounts in the exoskeleton of crustaceans, in the flesh of salmon and trout, and in other marine organisms as well [58]. Astaxhantin esters [59,60], β-criptoxanthin, α-carotene, β-carotene [61], meso-zeaxanthin [62], canthaxanthin, lutein, zeaxanthin, and crustacyanin [63] can also be found in this organisms at lower concentrations (Figure 2).

**Figure 3:**
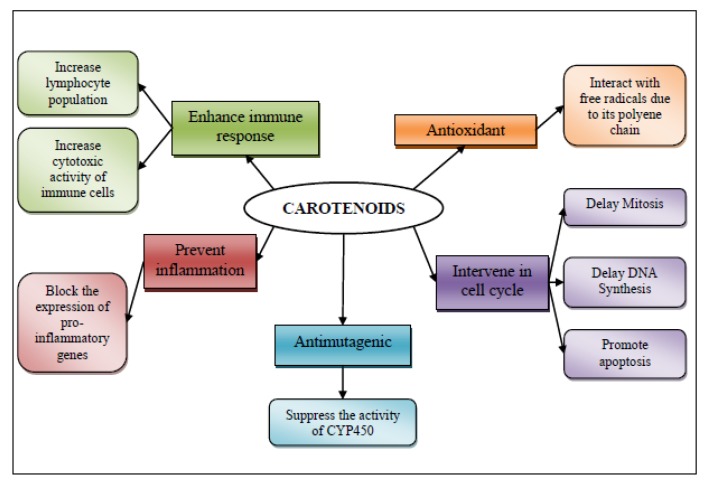
Mechanism of action for chemopreventive/chemoprotective activity of shrimp’s carotenoids.

Carotenoids have been associated to cancer prevention, which may undergo by five mechanisms of action: (1) membrane antioxidant [64]; (2) involvement in the control of cell differentiation and proliferation [65]; (3) antimutagenic effect; (4) as anti-inflammatory agents; and (5) their ability to produce an immune response in cancer (Figure 3).

#### 3.2.1. Antioxidant Mechanism

A number of studies have shown that carotenoids act as antioxidants by quenching singlet oxygen and free radicals [66]; this antioxidant activity directly emerges from the molecular structure of carotenoids [64], specifically due to the vibration of the polyene C=C and C–C bonds, where the energy of the singlet state oxygen is transferred to the carotenoid by direct contact [67,68].

The quenching activity of the different carotenoids mainly depends on the number of conjugated double bonds of their molecule and is influenced to a lesser extent by the functional end groups or by the nature of substituent in carotenoids containing cyclic groups [69]. In fact, carotenoids containing nine or more conjugated double bounds in their structure are potent singlet oxygen quenchers, a biological function which is independent of the provitamin A activity [70].

Astaxanthin and its esters, the main carotenoids present in the lipidic fraction of shrimp, have reported strong antioxidant activity in *in vitro* assays, as well as in membrane model system using phospholipid liposomes [7]. This carotenoid has a higher antioxidant activity than α-tocopherol, α-carotene, lutein, β-carotene, lycopene [71], and even higher than trolox [72], a synthetic antioxidant with a known high antioxidant activity. Martínez *et al.* (2008) [73] proposed the creation of a donor-acceptor map by measuring the electro-donating and the electron-accepting power; using this method, astaxanthin had the most effective antiradical effect in terms of its electro-acceptor capacity compared to other dietary carotenoids. This efficient antioxidant capacity is attributed to the unique structure of the terminal ring moiety. Due to these characteristics, astaxanthin may inhibit the production of lipid peroxides [74], as well as maintain the mitochondria in a reduced state even under oxidative challenge [75]. Liang *et al*. (2009) [48] suggested that astaxanthin scavenges radicals in the water/lipid interface and transfers an electron from a non-polar and more reducing carotenoid in the membrane. In cell cultures, astaxanthin has been able to act as an antioxidant even at high concentrations, when cells are exposed to oxidative stress [76]; however, other studies have reported pro-oxidant behavior in the same concentrations [77,78,79] this effect is reported when the experimental conditions include a low α-tocopherol diet, this compound usually helps carotenes to be regenerated; if there is an absence it can induce the formation of peroxyl radicals and or augment the rate of carotenoid auto-oxidation.

In *in vivo* studies, astaxanthin attenuates the promotion of hepatic metastasis induced by restraint stress in mice, throughout inhibition of the stress-induced lipid peroxidation [80]. In another study, when rats were exposed to mercuric chloride (a nephrotoxic compound that increases reactive oxygen species by decreasing glutathione levels due to its affinity to SH groups) and astaxanthin, the xantophyl was able prevent the increase of lipid and protein oxidation and attenuated histopathological changes [81].

Although these studies have proved the antioxidant activity of carotenoids, these compounds may shift into a pro-oxidant effect, depending on different factors such as oxygen tension or concentration. Mixtures of carotenoids alone or in association with others antioxidants may increase their activity against lipid peroxidation [69].

#### 3.2.2. Intervention in Cell Cycle

The cell cycle, a sequence of events by which a growing cell duplicates and divides into two identical daughter cells [82], involves numerous regulatory proteins that drive the cell throughout a sequence of specific events called cycle phases: G_1_, S, G_2_, and M [83]. Cells in G_1_ phase can, before commitment to DNA replication, enter into a resisting state called G_0_, the state where most non-growing and non-proliferating cells are in human body [84]. G_1_ and G_2_ phases are the “gaps” in the cell cycle that occur between the two landmarks, DNA synthesis (S) and mitosis (M); during G_1_ and G_2_ phases, the cell is preparing for DNA synthesis, and for mitosis, respectively [83].

Each of the cell cycle phases involves a number of proteins that regulate cell’s progression such as cyclin-dependent kinases (CDKs) and cyclin proteins [83,85]. Cancer cells are frequently associated with genetic or epigenetic alteration and these proteins help cells to sustain proliferation independent of external mitogenic or anti-mitogenic signals [84]; therefore, the regulation of the cell cycle may constitute a strategy to inhibit proliferation of cancer cells. It has been proposed that carotenoids affect differentiation and proliferation of cancerous cells. Different carotenoids (such as α-tocopherol, β-carotene, lycopene, and lutein) show different abilities to control cell cycle [86].

β-Carotene has been associated to both, cell apoptosis and inhibition of cell cycle throughout different mechanisms of action. In the cell cycle of normal human fibroblasts, β-carotene has a delaying effect on the G_1_ phase [87] which is related to the expression of p21^waf1/cip1^ protein (an inhibitor of CDK) [88]; in colon cancer, the presence of β-carotene has been associated to a reduced expression of cyclin A (regulator of G_2_/M progression) [89]; in leukemia cells, the inhibition of cell cycle progression is attributed to the up-regulation of PPARγ signaling pathway and the expression of Nrf2, an important transcription factor in Keap 1-Nnf2/EpRE/ARE signaling pathway [90]; therefore, the *in vitro* effect of β-carotene on the cell cycle depends on the studied cell line. On the other hand, β-carotene influences and enhances cellular apoptosis by modulating the expression of regulatory genes in cancer cell lines such as colon [89], leukemia [91], melanoma [92], and breast [93]. The mechanism of action is the suppression of apoptosis blocking proteins (specifically the protiens Bcl-2 and Bcl-xL) [91,92,93].

Astaxanthin has also been related to both, the inhibition of cell growth and apoptosis, in *in vitro* [94] and *in vivo* [95] studies. The apoptosis mechanism in hepatoma cell cancer has been attributed to depletion of GSH levels [94], and in leukemia cells to down-regulation of Bcl-2 protein [96]. The inhibition of cell cycle progression and apoptosis mechanisms are attributed to the up-regulation of PPARγ signaling pathway and the expression of Nrf2, an important transcription factor in Keap 1-Nnf2/EpRE/ARE signaling pathway [90].

Lycopene has been related to the insulin-like growth factor I (IGF-I); a factor that, at high blood levels, is related to breast and prostate cancer. This carotenoid changes the amount or affinity of IGF-I receptor signaling and cell cycle progression; therefore, lycopene has been related to the inhibition of cell progression at the G_1_ phase throughout the reduction of cyclin-dependent kinase (cdk4 and cdk2) [97] as well as the decrease in cyclin D1 and D3 [98]. In other *in vivo* studies including α-tocopherol, β-carotene, lycopene, and mixed carotenoids, in which they were used to treat cancer-induced hamsters, these carotenoids acted as suppressors of the cell cycle inhibiting the expressions of proliferating cell nuclear antigen (PCNA) and cyclin D_1_ [86].

#### 3.2.3. Antimutagenic Activity

Individual carotenoids such as astaxanthin and its esters, meso-zeaxanthin, β-carotene, zeaxanthin, α-carotene, among others, as well as their mixture, have been tested in the Ames test [99]. Researchers have found that these structures are able to produce an antimutagenic activity [99,100]. The inhibition of mutagenicity depends on both, the structure of the carotenoid and the mutagenic agent used.

Using sodium azide, ethidium bromide, and hydroxyl amine as control mutagens, astaxanthin and its esters, have shown high antimutagenic activity followed by lutein, β-carotene, violaxanthin, zeaxanthin, and α-carotene; however, a mixture of different carotenoids has shown higher inhibition of induced mutation in *Salmonella typhimurium* tester strains [99]. These results are supported by the study by González de Mejía *et al*. (1998) [100]; they assert that carotenoids have synergistic effect when the tester strains are exposed to 1-6-dinitropyrene (1,6-DNP) and 1,8-dinitropyrene (1,8-DNP) as control mutagens. However, not only those carotenoids have antimutagenic activity, meso-zeaxanthin has also proved biological activity when tested with sodium azide, 4-nitro-*O*-phenylenediane, and *N*-methyl-*N*′-nitro-*N*-nitrosoguanidine, as control mutagens. Meso-zeaxanthin showed 41 to 93% of mutagenesis inhibition in all *Salmonella* tester strains used [62]. β-Carotene also showed positive results when tested against 1-methyl-3-nitro-1-nitrosoguanidine and benzo[a]pyrene as control mutagens in *Salmonella typhimurium* TA100 tester strain [101].

Canthaxanthin and commercial carotene have been able to inhibit neoplastic transformation in cell culture exposed to the carcinogen 3-methylcloranthrene; this inhibition stopped after the removal of the carotenoid treatment [102].

In *in vivo* studies, the antimutagenic activity can be described as the ability of a compound to inhibit the mutagenic effect of a known mutagen in an animal model. In this sense, the mechanism of action for meso-zeaxanthin is the inhibition of CYP450 enzymes, which was demonstrated in a rat model, where even low concentrations of meso-zeaxanthin showed inhibitory effect towards various isoforms of CYP450 [63]. β-carotene has also proved *in vivo* activity in a Haffkine Swiss mouse tumor model [101], as well as in a Fisher 344 rats model [103] in which cancer was induced with *N*-ethyl-*N*-nitrosourea; the authors attributed the effect the unmetabolized β-carotene molecule, they concluded that this compound is absorbed, stored, and exerted antimutagenic effects against the chemical carcinogen without being transformed in the gastrointestinal tract.

#### 3.2.4. Anti-Inflammatory Mechanism and Tumor Immunity

The mechanism by which carotenoids enhance the immune system is still unclear. Studies have revealed that astaxanthin can prevent inflammatory processes by blocking the expression of pro-inflammatory genes, as a consequence of suppressing the nuclear factor kappaB (NF-κB) activation; moreover, carotenoids inhibits the production of nitric oxide and prostaglandin E2, and the pro-inflammatory cytokines tumor necrosis factor-alpha (TNF-R), as well as the interleukin-1 beta (IL-1β) [104]; this cytokines are molecules that mediate cell-to-cell interactions [105].

The immuno-regulatory action of carotenoids have been demonstrated through their role in tumor immunity [105]. These carotenoids enhance lymphocyte blastogenesis, increase the population of specific lymphocyte subsets, increase lymphocyte cytotoxic activity, and stimulate the production of various cytokines [106].

Wang *et al*. (1989) [107] demonstrated the inhibitory effect of carotenoids (beta-carotene, lycopene, and crocetin) on the growth and development of the C-6 glioma cells inoculated in rats, cells whose growth was inhibited in 57%–67% after 7 weeks without any observable hepatotoxic effect.

Lutein decreases H_2_O_2_ accumulation by scavenging superoxide and H_2_O_2_, and the NF-κB regulates inflammatory genes, iNOS, TNF-α, IL-1β, and cyclooxygenase-2, in lipopolysaccharide (LPS)-stimulated macrophages [108].

In other *in vivo* studies, dietary astaxanthin heightened immune response in domestic cats, which was attributed to the enhanced delayed-type of hypersensitivity response, peripheral blood mononuclear cell proliferation, natural killer cell cytotoxic activity, and increased T cell population [109]. In a similar way, dietary astaxanthin in dogs enhances lymphocyte proliferation, and natural killer (NK) cell cytotoxic activity; it also increases concentrations of immunoglobulin G and M (IgG and IgM), and B cell population. Therefore, dietary astaxanthin heightened cell-mediated and humoral immune response, and reduced DNA damage and inflammation in dogs [58,71]. In rats, astaxanthin was able to modulate the expression of NFkB, COX-2, MMPs-2/9, and ERK-2; therefore, it had an anti-inflammatory effect [95].

### 3.3. Polyunsaturated Fatty Acids

**Figure 4:**
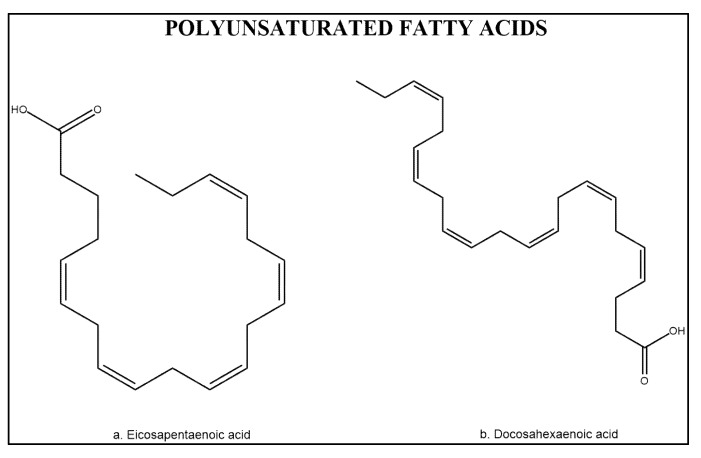
Chemical structure of polyunsaturated acids eicosapentaenoic acid (EPA) and docosahexaenoic acid (DHA).

Polyunsaturated fatty acids (PUFAs) in shrimp account for about 40% of the total fatty acid content [110] with about 15% occurring in the form of ω-3 and ω-6 fatty acids such as eicosapentaenoic acid (EPA) and docosahexaenoic acid (DHA) (Figure 4). Therefore, the quality of the fatty acid profile is similar to that of most of the marine fish species in terms of EPA and DHA content [1]. Both, fat and unsaturated fatty acids contents in shrimp muscle vary with diet [44], species [1], and maturity stage [111]. PUFAs are a subclass of bioactive components divided into two groups ω-6 and ω-3 fatty acids, both studied for cancer chemoprevention [112].

The polyunsaturated fatty acids have been identified to have a role in ameliorating various human diseases [113]. The pioneering epidemiological work on Greenland Inuit [114,115] suggested a possible link between the low incidence of heart diseases and the high consumption of seafood. Recently, the Women’s Intervention Nutrition Study (WINS) provided evidence that dietary lipids may influence local or distant recurrences, and in turn influence survivorship of woman in breast cancer treatment [116]. PUFAs uptake has also been inversely related to prostate [117], breast [118], and colorectal [119,120] cancer. Nevertheless, the association between fatty acid consumption and the reduction of cancer is still controversial. Some studies have related it with no effect [121,122] or even an increase in the risk for cancer [123], whereas clinical trials on the effect of polyunsaturated fatty acids are currently being conducted [124]. The inconsistent association observed in epidemiologic studies has been attributed mainly to three reasons [125,126], (1) the intake of the long-chain fatty acids used in the studies was too low to produce a protective effect; (2) in observational research, there has been weakness in estimating PUFAs consumption, mainly due to the difference in oil content between the different species of fish; and (3) the inclusion of low variability within-population in the intake of fish or ω-3 fatty acid.

Zhang *et al*. (2010) [127] proved three different oils diets containing ω-3, ω-6, and ω-9, suggesting that diets rich in ω-3 fatty acid oil attenuates the neoplastic effect of acrylamide-induced neoplasia in mice, while diets rich in ω-6 and ω-9 oils seemed to promote this activity.

**Figure 5:**
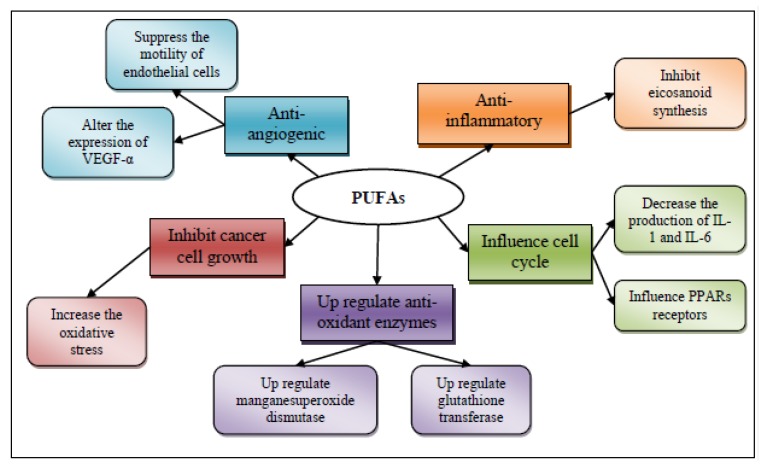
Mechanisms of action for the chemopreventive/chemoprotective activity of polyunsaturated fatty acids (PUFAs).

The mechanisms of action for the chemopreventive properties of ω-3 fatty acids are presumably five [125]: (1) suppression of arachidonic acid-derived eicosanoid biosynthesis [128]; (2) influence on transcription factor activity [129]; (3) increased or decreased production of free radicals and radical oxygen species; (4) mechanisms involving insulin sensitivity and membrane fluidity; and (5) antiangiogenic potential (Figure 5).

#### 3.3.1. Anti-Inflammatory Effect of Polyunsaturated Fatty Acids

As mentioned above, PUFAs have been associated to cancer chemoprevention through the inhibition of the arachidonic acid-derived eicosanoids formation. These compounds, characterized by 20 carbon long lipophilic molecules derived from arachidonic acid, are powerful regulators of cell function.

The generation process of eicosanoid compounds consists in a series of steps beginning with the mobilization of arachidonic acid from the cellular membrane glycerolipid pools by phospholipase A_2_ (PLA_2_). Then, arachidonic acid can be oxidized by three different enzymes: (1) the cyclooxigenases (COX-1 and COX-2) to form prostanglandins, protacyclin or tromboxanes; (2) lypoxigenase (LOX) to form 5(S)-, 8(S)-, 12(S)-, and 15(S)-hydroxyeicosatetraenic acid (HETE) leukotrienes, lipoxins, and hepoxilins; and (3) P450 epoxygenase (EOX) to form HETEs and epoxyeicosatrienoic acid (EET) [130].

The overexpression of eicosanoid-forming enzymes [COX, LOX, and prostaglandin E synthase (PGE)] has been related to cancer development in a wide variety of human and animal tumors [131]. These enzymes promote tumor proliferation and angiogenesis, and inhibit apoptosis [132]; for example, COX2 is normally absent in most cells, however, is highly induced in early stages of tumor progression [133].

Some studies have proved that PUFAs enhance the anti-inflammatory response in people with other conditions such as rheumatoid arthritis [134,135], and it has even been concluded that fatty acids can even be used as non-steroidal anti-inflammatory drug (NSAID) in patients with this pathology [136]. This type of drugs has been reported to be beneficial, since they reduce the risk of developing solid tumors in breast, colon, lung, and prostate cancers [137,138].

Gogos (1998) [139] carried out a randomized prospective study with patients with solid tumors who received fish oil supplementation; patients treated with ω-3 PUFA had an increased immunomodulating effect and prolonged survival.

The anti-inflammatory effect of PUFAs may also be attributed to their action on macrophages [140]; they decrease the production of IL-1, IL-6, and the tumor necrosis factor-α (TNF-α) when feeding ω-3 PUFA containing oil to rats [141].

#### 3.3.2. Influence in Transcription Factor Activity

According to Larsson *et al*. (2004) [125], one of the chemopreventive mechanisms of PUFAs is the modification of gene expression and signal transduction involved in the cell cycle. One of the transcription factor regulated by fatty acids is the peroxisome proliferator-activated receptors (PPARs), which are members of the nuclear hormone receptor family, the largest family of transcription factors [142]. Three PPAR have been identified including PPARα, PPARβ/δ, and PPARγ [143], all of them can be activated by naturally occurring fatty acids or fatty acid derivatives [144]. Their functions underlie a multitude of cellular and physiological processes by directly modulating target gene expression and indirectly modulating other transcription factors [142,143].

The effect of activating PPARβ/δ in cancer models and cancer cell lines depends on the cell line. Colon cancer is the most studied type of cancer and one of the proposed mechanisms of regulation is throughout the up-regulation of the adenomatous polyposis coli (APC)/β-CATENIN/transcription factor 4 (TCF4), pathway in which PPARβ/δ is activated by COX2-derived ligands (such as prostacyclins), leading to the expression of target genes that increase cell proliferation and promote tumor growth. Another proposed mechanism is the ligand activation of PPARβ/δ, which mediates terminal differentiation of colonocytes and inhibits cell proliferation [145]. Most fatty acids are considered PPARs agonist; nevertheless DHA suppresses its activation. This statement was demonstrated by Lee and Hwang (2002) [146] in colon tumor cells (HCT116).

#### 3.3.3. Increased or Decreased Production of Free Radicals and Radical Oxygen Species

Free radicals and reactive oxygen species (ROS) produced in cells seems to attack fatty acids present in the cell, in order to form a variety of by-products from lipid oxidation, including more free radicals and reactive aldehydes [50]. These metabolites potentially generate pro-mutagenic compounds, which eventually can lead to cancer development [147]. Nevertheless, highly polyunsaturated fatty acids, specifically EPA and DHA present in fish oil, have been proved to help up-regulate some antioxidant enzymes such as glutathione transferases and manganese superoxide dismutase in an *in vivo* study with mice [148].

The metabolites from the oxidation of *n*-3 PUFAs inhibit breast [149] and colon [150] cancer cells. This effect, observed in cell culture studies *in vitro*, was related to the formation of lipid peroxidation products, but the inhibitory effect is lost when they are exposed to vitamins that have antioxidant activity (specifically vitamin C and E [149]). Nevertheless, clinical trials have demonstrated that the DNA damage decreases with the intake of vitamin E when a high intake of PUFAs is taken [151].

#### 3.3.4. Antiangiogenic Potential

A high rate in neovascularization in solid tumors has been associated with a negative prognosis for cancer patients [152,153], since cancerous cells need the nutrients from the vascular system. Therefore, antiangiogenic agents may be helpful in cancer.

EPA [154] has proved *in vitro* antiangiogenic activity with a dose-dependent response for inhibition. PUFAs has also been used in an *in vivo* study, to prove an enhanced response of docetaxel (a drug used for antiangiogenic purposes in chemotherapy) with the aid of DHA [155], where both, micro and macrovascularization in the Sprague-Dawley rat model, were inhibited.

Two mechanisms have been suggested for the anti-angiogenic potential of PUFAs: the suppression of motility of endothelial cells [156], and alterations in the expression of the pro-angiogenic vascular endothelial growth factor (VEGF)-α [157].

3.4. ω-3 Fatty Acids as a Co-Treatment during Chemotherapy

In *in vitro* studies, DHA and/or EPA have been found to improve the cytotoxic effects of several anticancer drugs toward cell lines such as breast, colon, bladder, neuroblastoma, and glioblastoma [158,159]. This observation has also been made in animal models in different types of cancer such as lung, colon, mammary, and prostate [51]. The proposed mechanism of action for this beneficial effects is attributed to the change of the phospholipids in the cell membrane to more polyunsaturated fatty acids, mainly DHA and EPA; this alters the physical properties of the plasma membrane, resulting in an increase in membrane fluidity, enhancing the uptake of the chemotherapy drugs [160]. However, they can only be used at a maximum dose of 0.3 g/kg, according to a phase I clinical trial where adverse effects, mainly diarrhea and vomit, were observed [161].

Xenographic studies have been carried out to explain the benefits of a diet rich in PUFA’s in chemotherapy treatment. Atkinson *et al*. (1997) [162] inoculated fibrosarcoma tumor cells into Fisher 344 rats and fed them with diets containing different proportions of safflower oil and DHA oil, and treated them with arabinosylcytosine (AraC) or saline. Authors concluded that consumption of a diet rich in DHA could slow tumor growth, prevent hyperlipidemia, and enhance bone marrow cellularity, compared to a moderate-fat diet rich in ω-6 fatty acids. In a similar work, Cha *et al*. (2002) [163] investigated the effect of dietary supplementation with DHA in combination with AraC chemotherapy and found that DHA diet (1.8 g DHA/kg/day) can be associated with a longer life span and no incidence of death due to drug toxicity; nevertheless, the overconsumption of DHA (up to 4.5 g DHA/kg/day) shorten survival time, increases circulating tumor cell burden, and reduces red blood cell concentration.

The increased permeability of the small intestine of mice caused by methotrexate has been reported. Horie *et al*. (1998) [164] proved that oral administration of DHA protects the small intestine from the effects of methotrexate by reducing the permeability. Gomez de Segura *et al*. (2004) [165] studied the effect of DHA in male rats treated with 5-fluorouracil (5-FU), an antitumoral drug, and concluded that enriching diet with DHA protects the intestine from lesions produced by 5-FU.

In dogs with lymphoma, treated with doxorubicin chemotherapy in combination with PUFAs and arginine, Ogilvie *et al*. (2000) [166] observed that subjects with higher DHA plasma levels had better diet tolerance, and increased disease free interval and survival time.

A very specific example of the beneficial effects of ω-3 fatty acids was reported by Pardini *et al*. (2005) [167]. They reported that an old man diagnosed with malignant fibrous histiocytoma of the lungs, declined the conventional chemotherapy and elected nutritional intervention by changing his diet to a high ω-3 and low ω-6 supplementation. This study demonstrated that the size of the tumors was reduced, which was attributed to the intake of DHA, specifically to the ω-6/ω-3 ratio.

A proposed mechanism for the effect of the ω-3 fatty acids in chemotherapy is through the inhibition of the NF-κB transduction way, which suggests ω-3 PUFAs may be used during chemotherapy in cancer treatment [168].

## 4. Conclusions

There is an extensive research effort aimed to obtain efficient chemopreventive compounds in nature, mostly from vegetable sources. However, since the number of cancer cases is constantly increasing, the search, isolation, and study of chemopreventive compounds, has become an important area of research. Many of this research has focused on land organism; however, the great biodiversity that characterizes the marine environment, makes the search for bioactive compounds in this ecosystem a topic of great interest.

The lipidic fraction in shrimp is a source of chemopreventive compounds because its’ component, mainly attributed to carotenoids and PUFAs, have proved biological activity in both, *in vivo* and *in vitro* studies, as well as in xenographic research. Carotenoids exert their chemopreventive/chemoprotective activity mainly by four mechanisms: antioxidation, antiproliferation, antimutagenisis, and anti-inflammatory action, and these activities are mainly attributed to their chemical structure. On the other hand, PUFAs exert their chemopreventive potential mainly throughout four mechanisms: antiinflamatory and antiangiogenic activities, the ability to influence the transcription factor activity and the increased or decreased production of free radicals.

PUFAs could also be used as a co-treatment in cancer patients in order to enhance chemotherapy treatment as well as a chemopreventive agent without adverse toxic effects.

Based on the above, the lipidic fraction of shrimp represents an important commodity with high potential for the search of chemopreventive agents. However, in order to select the appropriate compound to be proposed as chemotherapeutic agent, a good knowledge is required concerning the pathways that each type of compound may modulate.
